# Design Innovation and Entrepreneurship Organization Based on Psychological Cognitiveness of the Space Narrative

**DOI:** 10.3389/fpsyg.2021.733828

**Published:** 2022-01-06

**Authors:** Jieming Hu, Xin Zhang

**Affiliations:** ^1^College of Fashion and Design, Donghua University, Shanghai, China; ^2^School of Humanities, Shanghai Normal University, Shanghai, China

**Keywords:** design innovation, entrepreneurship, organizational culture, psychological cognition, spatial narrative, community of practice

## Abstract

The high-quality workspace can be used as a physical carrier for design innovation and entrepreneurial organizational culture to continuously change the psychological cognition and behavior of employees in community of practice. The spatial narrative of the culture of design innovation and entrepreneurial organizations means to integrate entrepreneurship and organizational culture into the space through visual presentation. Whether the spatial narrative is successful or not needs to be judged by whether the change of people’s psychological cognition achieves the expected effect. The traditional qualitative research methods such as interviews and questionnaires cannot fully and accurately present the psychological cognitive mechanism of design Innovation and entrepreneurship organization members. We use virtual reality technology combined with electrophysiological technology to conduct experiments. We use these technologies to conduct quantitative experiments on psychological cognition in community of practice. This study will select a design innovation and entrepreneurial organization, randomly select 20 participants, and divide them into 2 groups for experimentation. The VR scene is based on their real office space as a prototype. Put the visual elements of corporate culture in one of the VR scenes. The other VR scene as a reference does not incorporate visual elements of organizational culture. Participants participated in the experiment in these two VR scenarios. There are many advanced devices that can accurately test individual psychological changes, but the ErgoLab man-machine environment test platform, can collect and compare these data [physiological data, electroencephalogram (EEG) data, and behavior data] in real-time and comprehensively, which is its advantage. According to the experimental results, judge the changes in the psychological cognitive data of the participants before and after the placement of the spatial narrative in design innovation and entrepreneurial organizations. The experiment combined interviews and questionnaires to ensure the authenticity of the quantitative data. The conclusion of the experiment will produce an accurate quantitative study on the psychological cognition of the spatial narrative of design innovation and entrepreneurial organizational culture. A sense of organizational belonging, collective sense, pride, mission, and work fun can be generated in the workspace.

## Research Background, Trends and Significance

### Research Background

Early researchers in this field discovered that architectural space is closely related to storytelling. Architecture is permeated with narratives because it is constituted within a field of discourses and economies (formal, psychological, and ideological) (Rakatansky, 1992). The spatial narrative of architecture is closely related to the psychological changes of people. Narrative design, as the origin of post-modern, has expression function besides meeting the practicality demands (Xiang, 2005). This expression function can affect the psychological cognition of space users. Using narrative criteria to predict or interpret possible human response to interior design can assist designers in making wise concrete decisions about complex and abstract phenomena (Cathy and Ganoe, 1999). Researchers can use VR indoor scenes to test human cognition and behavior. The interior design of virtual reality can make designers and users realize the effect of “what you see is what you get,” and at the same time, make users feel the artistic conception in the virtual scene of this design (Li et al., 2021). VR had been applied as a visualization tool to construct a dynamic and immersion environment that imitates the campus space. Then some key design elements were changed to form contrast schemes. Participants were recruited to experience these contrast spaces and were collected Electrodermal Activity (EDA) and Photoplethysmography (PPG) signals for emotional arousal analysis (Pei et al., 2021b). Davies (2010) believed that Office design needs to be based on the needs of the most important producers of profit and value for any organization – the workforce. And ideas that office design can impact organizational culture – resulting in the adoption of more collaborative working spaces in trying to force interaction (Davies, 2010). However, in these studies, few people pay attention to how to influence the innovation enthusiasm of employees through the visual presentation of corporate culture in the office space. In these studies, no one paid much attention to the psychological impact of office space narratives on employees of entrepreneurial organizations. The development of design innovation and entrepreneurship requires a workspace that can stimulate employees’ creativity, gather team strength, and be full of corporate culture as a physical carrier. The team’s effectiveness can be improved by changing the space environment where the team works (Plattner et al., 2019). Differences in innovation capacity across different functional areas are impacted more by the Attitudes Toward Innovation that individuals bring to the workplace (Cropley and Cropley, 2017). From the perspective of cultural psychology, the workspace can be used as a physical carrier for designing entrepreneurial organizational culture to continuously change employees’ psychological awareness and cognitive behavior, and generate a sense of organizational belonging. In order to find the modes of professionalization employed in the field of creative industries, the category of space and place have been addressed as a fruitful research area intended to contextualize the sometimes self-driven evolution? in creating an identity as an entrepreneur (Lange, 2011). Design is a part of art and needs creative inspiration. One of the main motivations expressed in the academic literature in the emerging field of artistic entrepreneurship is the need to improve the entrepreneurial skills of artists (Callander and Cummings, 2020). The spatial narrative of designing innovation and entrepreneurial organization culture is to integrate entrepreneurship and corporate culture symbols into the space through visual presentation. Dissemination of unique corporate cultural information through specific spatial visual logic, so as to use space to “tell” entrepreneurial stories, cultivate entrepreneurial spirit, stimulate creativity, and generate positive organizational psychology. Entrepreneurs attempt to contextualize innovation by establishing links with the past, present and future to generate meaning (Garud et al., 2014). This type of scientific research (environmental psychology) that triggers psychological changes through spatial cues is increasingly being paid attention to by design entrepreneurial organization teams and related scholars. Related studies have shown that placing ornamental plants in the office space will increase the happiness of employees. The researchers used quantitative research methods to test participants’ brain activity, heart rate variability, and skin conductivity. Finally, the researchers found Ornamental flowering plants are beneficial to improve the physiological functions of office workers and improve psychological relaxation (Elsadek and Liu, 2020). However, there are relatively few related researches on the precise quantitative research on the psychological cognition of spatial narrative in the design of entrepreneurial organization culture, and it is worthy of in-depth study.

Therefore, we try to re-understand the psychological cognition of spatial narrative in designing entrepreneurial organizational culture through scientific and precise quantitative research in this paper. The purpose of this paper mainly contains three important contents. The first important content is to change the situation of insufficient precision quantification in the research of spatial narrative psychology in the design of entrepreneurial organization culture through this research. The second important content is that we try to establish a scientific method that can combine qualitative research and quantitative research in this research field through this paper. The third important content is that we try to establish a scientific evaluation of spatial narrative psychology from multiple dimensions. The main structure of this article is based on academic research analysis and practical needs to ask questions, then carry out experimental design and data collection, and finally carry out data analysis. We use these data to try to solve the problems raised.

### Research Trends

**FIGURE 1:**
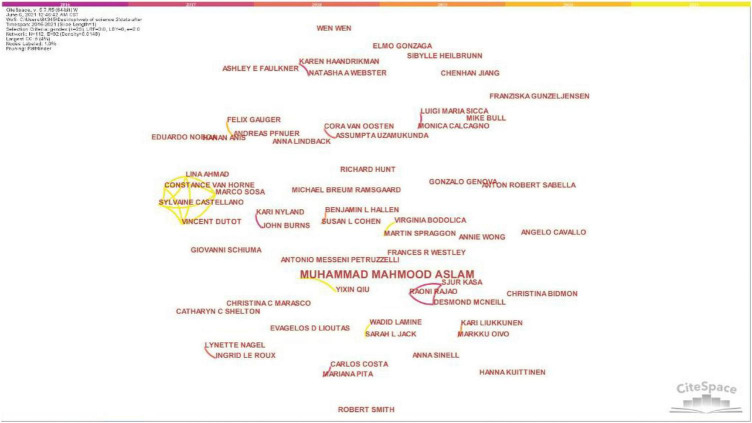
The analysis of the author’s knowledge map drawn by CiteSpace (Retrieved by our research team and screenshots).

The research team selected the world academic literature abstract index database Web of Science to retrieve and analyze the content of the research on the design, innovation and entrepreneurship organization space. Under the selected database of the core collection of Web of Science, the three subject terms “design,” “entrepreneurship” and “space” were selected for retrieval. The publication time was set to nearly 5 years. A total of 71 related journal articles were searched. In order to visualize the state of this research, the research team imported the retrieved data into the CiteSpace bibliometric analysis tool to analyze the original data, and drew relevant knowledge maps from the three aspects of the author, research institution, and keywords for visual analysis. Through the analysis of the author’s knowledge map drawn by CiteSpace (Figure 1), it is found that the correlation degree of the researchers of “design,” “entrepreneurship” and “space” shows a total of 112 nodes, showing 92 connections, and the correlation density is only 0.0148. These data indicate that the closeness of the association between researcher’ meetings is low; the research frequency is less, and the research in this field is not sufficient, and it is worth continuing to study.

**FIGURE 2:**
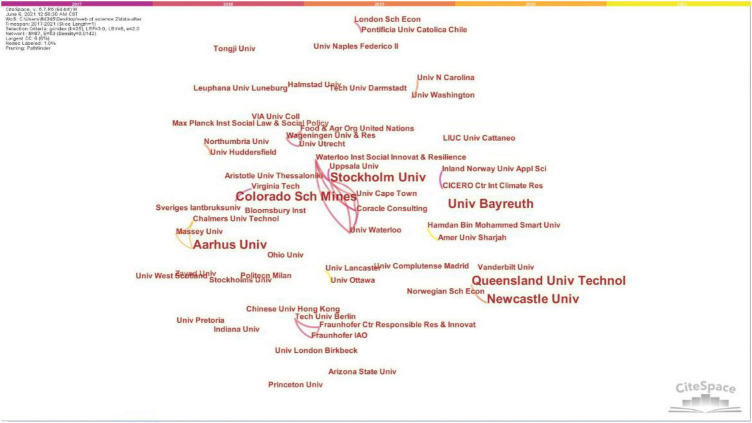
The analysis of the knowledge map of research institutions drawn by CiteSpace (Retrieved by our research team and screenshots).

Through the analysis of the knowledge map of research institutions drawn by CiteSpace (Figure 2) and related background data, it is found that the cooperation status of research institutions for “design,” “entrepreneurship,” and “space” is scattered. According to the knowledge graph, the co-occurrence network of research institutions shows 87 nodes, 53 connections, and the density is only 0.0142. Although some research universities cooperate more with other research institutions, the overall research strength is scattered, and the research in this field is relatively weak. Stockholm University in Sweden has many connections and has the most research institutions in cooperation with it. It has connections with universities and research institutions such as Waterloo Institute for Social Innovation and Resilience, Uppsala University, University of Cape Town, Coracle Consulting, and University of Waterloo.

**FIGURE 3:**
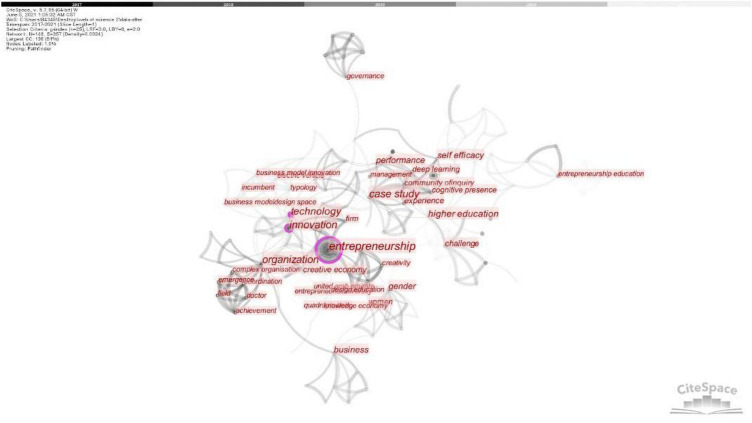
The keyword knowledge map drawn by CiteSpace (Retrieved by our research team and screenshots).

Through the analysis of the keyword knowledge map (Figure 3) drawn by CiteSpace, the frequency and centrality of some keywords can be counted (Table 1). The co-occurrence network and frequency analysis of keywords can detect relevant hot spots in this research. After importing the retrieved literature data, set “Time Slicing” to “2017–2021,” take 1 year as a time slice, and click “keyword” as the analysis object. Select the co-occurrence word TOP25% in each slice as the threshold, and then generate a keyword co-occurrence map for the research field of this problem from 2017 to 2021. Around the core keywords of “entrepreneurship,” co-occurring keywords such as “Innovation,” “Organization,” “Technology,” and “Case study” appeared. From the perspective of the frequency of keywords, the frequency of the main keyword “entrepreneurship” is 27, and the centrality is 0.49. The frequency of “Innovation” is 14, and the centrality is 0.23. The frequency of “Organization” is 6, and the centrality is 0.32. Although the overall centrality is low, entrepreneurship, innovation, and organization are the main topics discussed. Although “self efficacy” involves the field of psychology to a certain extent, there is a lack of research on environmental psychology for designing innovative and entrepreneurial organizational culture, so further research can be done.

**TABLE 1:** Keyword frequency and centrality statistics (top 20) (Retrieved, screenshots and sorted by our research team).

Serial number	Key words	Frequency	Centrality
1	entrepreneurship	27	0.49
2	innovation	14	0.23
3	organization	6	0.32
4	policy	6	0.08
5	technology	6	0.40
6	experience	5	0.07
7	entrepreneurship education	5	0.08
8	design	4	0.04
9	case study	4	0.54
10	business	4	0.15
11	self efficacy	4	0.24
12	strategy	4	0.17
13	performance	4	0.06
14	model	4	0.02
15	gender	4	0.21
16	creativity	3	0.10
17	firm	3	0.12
18	discourse	3	0.00
19	enterprise	3	0.04
20	higher education	3	0.15

### The Innovation and Effectiveness of the Research

Whether the study of spatial narrative is successful needs to be judged by whether the change of people’s psychological cognition achieves the expected effect. The traditional qualitative research methods such as interviews and questionnaires cannot fully and accurately present the psychological cognitive mechanism of design innovation and entrepreneurial organization members. Moreover, the experimental process of a lot of related research is not multi-dimensional, real-time comprehensive data collection and analysis. Therefore, we used a new technology platform and test methods to conduct experiments.

Regarding this research, we have two important innovations. First, there are innovations in research methods in this research. Our experiments are tested in the created 3D virtual reality environment. Through multi-dimensional objective and quantitative experimental data analysis, combined with the process of qualitative research, we make the research method more scientific and comprehensive. Our exploration in research methods makes up for the lack of precise quantitative research in this field. We use ErgoLAB HME Synchronization Technology to collect the physiological data, electroencephalogram (EEG) data and eye movement data of the testees to realize the real-time synchronous collection and analysis of multi-dimensional data. Therefore, the results of this experiment are more accurate. Second, there are new research findings in this research. Through this research, we have revealed the subtle changes between the spatial narrative and individual psychological cognition about the design and innovation of entrepreneurial organizational culture. We have confirmed through experiments that the implantation of entrepreneurial organizational culture has a positive impact on people’s psychological cognition in the process of spatial narration. An office space that incorporates design innovation and entrepreneurial organizational culture is conducive to inspiring employees’ sense of belonging, collective sense, pride, mission, and work fun.

In this study, we selected a real design innovation and entrepreneurship organization as the experimental object, and randomly selected subjects from the organization for experimentation. Therefore, it is effective in the selection of experimental subjects. We use the combination of quantitative and qualitative research methods, and use the more advanced ErgoLAB HME Synchronization Technology in the research. Therefore, our experimental methods and techniques are effective. In the process of research, we draw a visual map based on CiteSpace and conduct a lot of literature research. Therefore, there is a certain academic basis in this research. This research has three important values. First, through a combination of quantitative research and subjective evaluation, we empirically demonstrated that using the organizational culture of design innovation and entrepreneurship as a spatial narrative content will have a positive impact on human psychology. Second, we obtain data through scientific quantitative experiments, which improves the scientificity and accuracy of the spatial narrative process of entrepreneurial organizational culture. The results of this experiment provide a data reference for stimulating the innovation ability of members in innovative and entrepreneurial organizations. Third, we explored the specific content of the psychological impact of spatial narration on the employees of design innovation and entrepreneurial organizations. The office space integrated into the design, innovation and entrepreneurship organization culture is conducive to inspiring employees’ sense of belonging, collective sense, pride, mission, and work fun. People who design innovative and entrepreneurial organizations can use these dimensions to carry out spatial narration in the workspace, and to achieve the harmony and unity of people, culture, and environment.

It is worth exploring whether the integration of organizational culture into the office space has an impact on employees enthusiasm for work. This research mainly focuses on the influence of organizational culture on employees’ sense of belonging, collective sense, pride, sense of mission, and sense of work fun to form judgments. An excellent organizational culture should give employees a sense of belonging, which is also one of the important manifestations of the formation of organizational identity, trust and emotional maintenance. The sense of collectiveness plays an important role in the teamwork and efficient operation of the members in the organization, which can enhance the passion at work and help to form a common goal. Pride has an important impact on the self-confidence development of members in an organization, and can increase job satisfaction. The sense of mission forms a consistent value cognition to the sense of responsibility and responsibility of the members in the organization. The sense of fun at work plays an important role in the happiness index, thinking transformation, happy mood of the members in the organization and alleviating work fatigue.

## The Experimental Content of the Research

### Research Hypothesis and Experimental Preparation

The research hypothesis proposed by our team is that the implantation of design Innovation and entrepreneurial organizational culture in the office space will have a positive impact on people’s psychological cognition during the spatial narrative process. The office space integrated into the organizational culture of innovation and entrepreneurship is conducive to inspiring employees’ sense of belonging, collective sense, pride, mission, and work fun. Entrepreneurial organizational culture is very important to the development of a company. Teams whose members have a clear focus on their purpose within the organization’s over-arching strategic plans, are well matched with specific talents of members and leaders of the team, and serve within a strong enterprise culture, are more highly motivated as agents of making strategy happen (Wolf and Felger, 2019). The culture of entrepreneurial organization has an important influence on the employee’s job satisfaction and work motivation. Organizational Culture (OC) has a positive and significant effect on Employee Performance (EP) (Prawinto, 2020). Studies have shown that Organizational culture has positive influence directly and indirectly to the work satisfaction and organizational commitment. The work satisfaction has positive influence directly or indirectly toward motivation and organizational commitment (Tobing and Kennedy, 2018). The development of design innovation and entrepreneurial organizations is inseparable from the support of workplace. Workplace design, configuration and spatial features impact how well and how much a company can benefit from its human capital (Lu, 2015). In J. Lu’s paper, the sense of belonging should be a method of establishing connections with other elements in the office (Lu, 2015). Stress management, personal control, sense of belonging, territoriality, control and supervision are the identifiable dimensions that influence occupants’ innovation and collaboration, well-being and job satisfaction in the office environment (Nag, 2019). Therefore, the sense of belonging of employees should be paid attention to in the office space. Team workspace is a specific example of an alternative office design strategy to support the activities of highly interactive, multi-disciplinary teams of knowledge workers (Brager et al., 2012). Therefore, the collective sense of employees should be paid attention to in the office space. Y. Lu and V. Roto believe that pride is one of the most meaningful experiences in daily life. And Taking the interdisciplinary aspects of pride into account, their article addresses the challenge of how experience design can contribute to pride experience in the workplace (Lu and Roto, 2016). Therefore, the pride of employees should be paid attention to in the office space. The sense of mission can be a powerful force in shaping and guiding an entrepreneurial venture (Wickham, 1997). Therefore, the employees’ sense of mission should be paid attention to in the office space. R. Fluegge-Woolf found that Fun at work was positively and directly related to organizational citizenship behavior, and positively and indirectly to both task performance and creative performance. In addition, individuals having fun at work were also more likely to be more engaged in their work, and consequently exhibit greater creative performance (Fluegge-Woolf, 2014). Therefore, in the office space, attention should be paid to employees’ work fun.

Through the use of virtual reality imaging technology combined with electrophysiological technology to conduct quantitative experiments on the psychological cognition of the participants in the spatial narrative of the innovation and entrepreneurial organization culture. The main experimental equipment includes physiological index test equipment, EEG index test equipment, eye tracker and ErgoLab platform^1^, etc. The ErgoLab man-machine environment test cloud platform version 3.0 used in this experiment was provided by Beijing Jinfa Technology Co., Ltd. (Kingfar International Inc.). The independent variables of the experiment are mainly the sense of belonging, collective sense, pride, mission and work interest of the design innovation and entrepreneurship organization’s members. The dependent variables are mainly the physiological indicators, EEG indicators and eye movement indicators of the participants. Through experimental empirical research, it is clear whether the organizational culture of design innovation and entrepreneurship has an impact on people’s psychological cognition in the process of spatial narration.

The design innovation organization selected for this research is Shanghai Fengyuzhu Culture Technology Co., Ltd., which was established in 2003. From a small design innovation and entrepreneurial organization at the beginning of its establishment, it has now developed into a leading enterprise in China’s exhibition and display industry, mainly focusing on the exhibition and display design industry. This design organization has a compound design building with its own property rights and was officially listed in 2017. The main reason why this design innovation and entrepreneurship organization is selected as the research sample is that it can develop from a small design innovation and entrepreneurship organization to a leading enterprise in the industry in a relatively short period of time, and the organization development is distinctively typical. The organizational culture of this design organization is clear and systematic, with direct visual logos and slogans, and the development of the company is also clear. Another characteristic of this company is to focus on the creation of a high-quality office environment. The office space on each floor is equipped with a variety of scenic spots, leisure areas and interactive exhibition areas, as well as a botanical garden, gymnasium, coffee bar, indoor basketball court, 5D cinema, football field, virtual golf and other spaces and architectural model museums. These factors make the office space appear very comfortable and full of interest, and mobilize the enthusiasm of employees for innovation in a relaxed and pleasant environment. Just like the slogan of this design innovation and entrepreneurship organization – “The fun is just beginning,” this also highlights the difference in design innovation and entrepreneurship organizations.

The stimulus materials used in this experiment are two VR virtual office spaces made with computers. The structure, area and layout of these two VR virtual office spaces are basically the same. And it is a VR scene based on the real scene in their real work. The reason why VR scenes are used for experiments, because it helps minimize external interference factors during the experiment, such as interference caused by changes in light or other changing factors in the space. The VR scene is an open office area that can accommodate about 18 employees, with an area of about 148 square meters, and the environment is relatively high-quality. The difference is that the logo of the design entrepreneurial organization, slogan, corporate honors and awards display, corporate history memorabilia display, board game scene, team building activity display and other corporate cultural visual images are implanted in a scene space (scene B) and symbol. We take these visual elements that represent corporate culture as important content that can reflect spatial narratives. In another space (Scene A), the above visual content is not implanted. Test the changes in physiological indicators, EEG indicators and eye movements of the two groups of people under different environments. The VR virtual office space is re-established based on the real office scene of this design innovation and entrepreneurial organization. The original basic structure and basic layout of the space are retained in the virtual scene to ensure that employees do not have a complete sense of strangeness to the space. In the office space scene where the design and entrepreneurial organizational culture is implanted, we have carried out some back-end parameter associations to facilitate the use of eye trackers to analyze and capture later in the test process. In the VR virtual space, the logo is the most intuitive attribution mark of the entrepreneurial organization, so we associate the sense of belonging by setting the corporate logo image. The slogan of the organization can enhance the collective consensus, and shouting slogans is also an important means to strengthen the sense of collectiveness. Therefore, we set up the slogan slogan display to associate the sense of collectiveness. The honor of the enterprise can bring full confidence to employees, and it is also a reward for their own work. Therefore, we set up a display area for the honor of the enterprise to associate the sense of pride. Understanding the turning point of the company can motivate employees’ sense of responsibility, and understanding the company’s history is conducive to urging employees to shoulder their missions. Therefore, we set up a corporate history memorabilia display area to associate the sense of mission. The entertainment space allows employees to relax and entertain after work. The photos of team building activities can also witness the company’s daily fun life. Therefore, we set up board games and team building activity scene areas to associate work fun. Through the test of these five senses, it is possible to discover the specific degree of influence of corporate culture into the office space on employees.

### Experimental Operation Process

**FIGURE 4:**
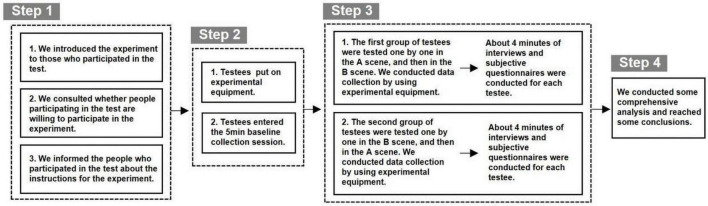
Schematic diagram of the process of this experiment (Drawn by our research team).

**FIGURE 5:**
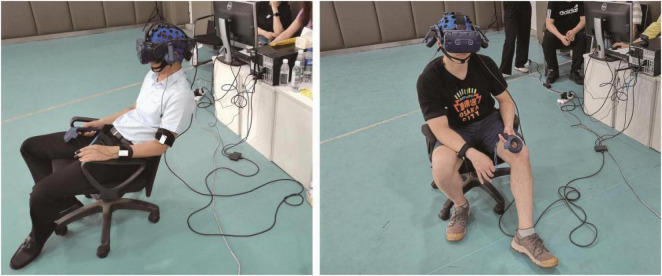
Image recording of field experiments on testees (Photography by our research team at the experimental site).

Through thorough communication with this design, innovation and entrepreneurship organization (Shanghai Fengyuzhu Culture Technology Co., Ltd.), we randomly selected 20 participants. Two groups (10 people in each group) conducted individual participant experiments in a space that did not belong to their entrepreneurial culture and a space that belonged to their corporate culture. Our experimental process is mainly divided into four steps(Figure 4). In the first step, we introduced the purpose of this experiment to the people who participated in the test, and entered the experiment after seeking their own consent. In the second step, the tested wear physiological data collection equipment, EEG data collection equipment, and eye movement data collection equipment according to the experimental arrangement, and enters the 5-min baseline collection link. In the third step, the testees enter the free browsing link of the VR office scene (Figure 5). Among them, the first group of testees (10 people) first look at the A space (office space without corporate culture implantation) for the VR test. The test time is 3 min, and various indicators are recorded at the same time in the background. Then, the subjects looked at Space B (office space with corporate culture implanted) for VR testing. The test time per person was 3 min, and various indicators were recorded at the same time in the background. The second group of subjects (10 people) was tested in the same format and time as the first group. The difference was that the second group of subjects looked at the B space first, and then looked at the A space. In this way, it can be ensured that there is no pre-effect in the experimental results of the test subjects in the experience of the two VR office scenes. In the process of two groups of people being tested in turn, we used ErgoLab eye tracking analysis and VR virtual eye tracking module to record and analyze the attention of the testees in real time. We use ErgoLab, man-machine environment test platform to collect eye movement data, physiological data, EEG data and behavior data for comprehensive analysis. During the test, the synchronized analysis of the eye movement parameters are the first fixation time (TFF) and the total fixation time. The physiological recording module records and analyzes the subjects’ physical arousal level, changes in stress response levels and emotional relief in real time, mainly using electrical skin activity (EDA) and ear tip pulse (PPG). The EEG recording module records and analyzes the brain waves of the subject in a awake, calm state, and an active and agitated state in real time. The synchronization analysis indicators are α waves and β waves. According to the experimental results, we judge the changes in the psychological cognitive data of the participants before and after the placement of the spatial narrative in the design of the innovation and entrepreneurial organization culture. After the virtual experience experiment is over, a 4-min subjective feeling interview and subjective questionnaire record module. The source of the survey questionnaire is the questionnaire design carried out by the research team around the employee’s sense of belonging, collective sense, pride, mission, and work fun in this research. Finally, a comprehensive analysis is carried out to ensure the authenticity of the quantitative data through a combination of qualitative and quantitative research.

## Analysis of Experimental Statistics

### Analysis of the Baseline Situation of the Testees

At the beginning of the experimental test, each testee needs to accept a baseline test in a calm state for 5 min. The baseline test is mainly to test the electrical skin activity (EDA), brain electrical indicators (EEG) and Heart Rate Variability (HRV) of testees. Electrical skin activity (EDA) is to determine the state of a person’s mental activity by testing the electrical conductivity of the skin. Emotional tension, excitement, excitement, and anxiety will all lead to an increase in skin conductance (Mandryk et al., 2006). Electroencephalogram (EEG) is mainly to test the brain wave changes of a person in different states. Brain waves can be divided into α waves, β waves, θ waves and δ waves. The frequency of α waves is 8–13 times per second, the average number is about 10 times, and the amplitude is 20–100 μV. The rhythm is most pronounced when the eyes are opened or when other stimuli are received, the α wave disappears instantly (Tie and Hui, 2007). The appearance of β waves means that the brain is more excited (Tie and Hui, 2007). Heart Rate Variability (HRV) signal contains a lot of information about cardiovascular regulation. The extraction and analysis of this information can quantitatively evaluate the tension and balance of cardiac sympathetic nerve and vagus nerve activity and its influence on cardiovascular system activity (Jingxia and Yanqiang, 2001).

After processing and exporting the data through ErgoLab data, it was found that the average value of the overall electrical skin activity (EDA) of the 20 testees was at a relatively low level. Except for very few samples, such as the first testee, the average EDA value was as high as 16.98 μS, and most of the EDA was below 10 μS. The overall average of the EDA of all samples is 3.22 μS, which indicates that most of the testees are in a relatively stable state of mind, and they have a better psychological adaptation to the tested environment.

Electroencephalogram baseline situation: research data show that alpha waves appear when awake, quiet, and closed eyes, and the alpha waves recorded in the back of the brain are most obvious, and when the eyes are opened, thinking problems or receiving other stimuli, The alpha wave disappears (Songyun et al., 2008). Therefore, alpha waves can be used to analyze the relaxed and comfortable mood of the testees in different scenes. Analyze the excitement of the testees in different scenes with β waves. After the research team processed and exported the data through ErgoLab, the alpha wave was selected as the analysis index in the EEG analysis. The overall total power average was 19.96 dB and the standard deviation was 12.07 dB. The average value of the total power of the β wave is 16.7 dB, and the standard deviation is 11.98 dB. Most of the subjects are conscious, their bodies are in a relaxed state, and the overall subjects are in good condition.

Heart rate variability (HRV) baseline situation: After processing and exporting data through ErgoLAB, meanHR (bpm) is selected as the analysis index in the time domain analysis of heart rate variability (HRV). Overall, the data shows that the average heart rate of the ECG signal is 77.65 bpm, and the samples are all between 60–100 bpm. In the frequency domain index analysis, LF/HF is selected as the analysis index, and the overall LF/HF ratio is basically between 0 and 5. After removing the ratio of sample 8 to 31 (which may be an abnormality caused by improper operation of the experiment), the overall average is 1.27. The overall signal power density is stable.

### Physiological Data Analysis of the Testees

**FIGURE 6:**
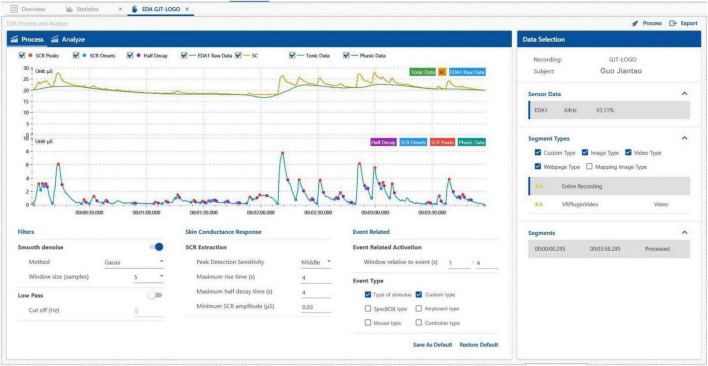
EDA indicator records of individual samples displayed on the ErgoLAB platform during the experiment (Search and take screenshots from ErgoLAB by our research team).

Electrodermal activity data analysis: Electric skin (EDA) baseline situation: The EDA indicator record of individual samples displayed on the ErgoLab platform during the experiment is shown in the figure (Figure 6). This figure shows the changes in the skin’s electrical conductivity data during the testee’s experience of scene B. An increase in the value during a certain period of time indicates that the testee’s emotional arousal is increased. It shows that the testee was stimulated by an event during this time period. When combined with the comprehensive analysis of the VR scene graph that the testee saw at the time, we can determine which factors caused this situation. After data processing and data export, it is found that the average EDA of the testees in scene A is 7.82 μS. Compared to the baseline EDA average of 3.22 μS, which is higher than 4.6 μS, this indicates that the testees were watching scene A and his mentality is still slightly fluctuating. Part of the reason may be due to the excitement and novelty of the testees wearing VR glasses.

After data processing and data export, it is found that the average EDA of the subjects in scene B is 7.9 μS, which is only 0.8 μS higher than the average EDA of scene A of 7.82 μS. Therefore, although the EDA data is not enough to show that scene B has a more obvious psychological impact on the testee for scene A. But objectively, it shows that the mentality of the testees when watching the two scenes is relatively stable, and other physiological indicators can be further analyzed.

Heart rate variability data analysis: time-domain analysis of HRV, the average heart rate of the overall ECG signal of the subject in scene A is 78.85 bpm. Compared with the baseline average heart rate value of 77.65 bpm, the overall increase is 1.2 bpm. Although the increase in this value is not obvious, it also shows that the testee’s heartbeat speeds up while observing the scene, and the degree of emotional arousal increases slightly.

The average heart rate value of the overall ECG signal of the testee in scene B is 79.75 bpm, which is higher than the average heart rate value of 78.85 bpm in scene A, and is 0.9 bpm higher overall, with a slight increase. The data shows that the testee’s heartbeat speeds up slightly when observing a scene with corporate culture space. The degree of emotional arousal has increased to a certain extent, and the overall degree of autonomic nervous system has increased. In the frequency domain analysis, the average LF/HF index of the testees in scene A is 2.02. Compared to the baseline LF/HF average of 1.27, an increase of 0.75. The data shows that the testee responds to an increase in the equilibrium control of the autonomic nervous system, which is a normal phenomenon.

The average LF/HF index of testees in scenario B is 3.73, which is 1.71- higher than the average of LF/HF in scenario A, an increase of 84%. The data showed that the testees responded that the balance control of the autonomic nervous system was significantly increased. It shows that the testees are more autonomous and proactive when observing the scene of the space with corporate culture implantation. At the same time, the overall average value of β waves in scene A is 34 dB, and the overall average value of β waves in scene B is 35.61 dB. Compared with the baseline, the overall average value of β wave is 16.7 dB, and the value in scene B is the highest, indicating that some of the testees in scene B are more exciting.

### Electroencephalogram Data Analysis of Testees

**FIGURE 7:**
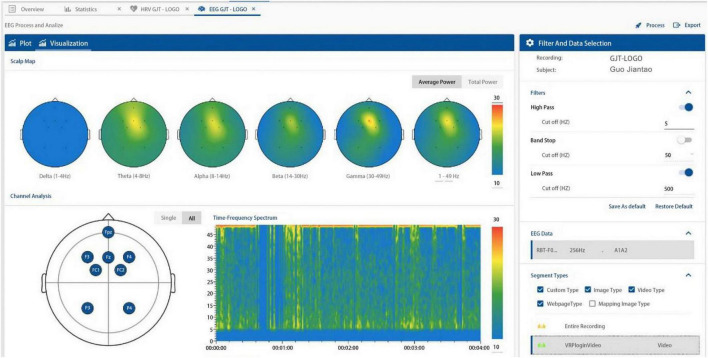
EEG indicator records of individual samples displayed on the ErgoLAB platform during the experiment (Search and take screenshots from ErgoLAB by our research team).

**FIGURE 8:**
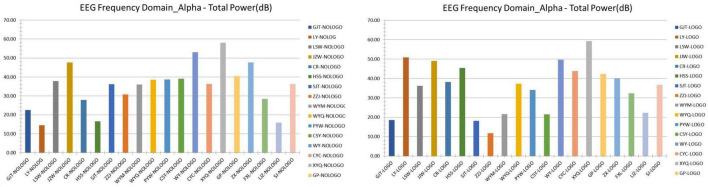
EEGα wave of the testee in scenes A and B-total power (dB) (Search and take screenshots from ErgoLab by our research team).

The EEG indicator record of individual samples displayed on the ErgoLAB platform during the experiment is shown in the figure (Figure 7). The figure reflects the brain wave changes of the testee when the test is performed in scene B. According to this graph, we can see the brain excitement of the testee in different test time periods. When the excitement is high, it means that the testee may be stimulated by some images in the scene to cause changes in brain waves. We first analyzed the alpha wave (Figure 8). After data processing and exporting the data, the analysis found that the overall average value of the alpha wave in the EEG indicators of the testee in scene A (9–14 Hz) was 35.15 dB, compared with The overall average value of the alpha wave from the baseline is 19.96 dB, an increase of 15.19 dB. This shows that the testee’s awareness has increased significantly under scenario A and the baseline test. In scene B, the overall average value of the alpha wave of the testee is 35.48 dB, which is 0.33 dB higher than that of the testee in scene A, and the value has increased. This shows that although the level of consciousness of the testees in the two scenes is basically the same, scene B is more comfortable for the testees.

### Eye Movement Data Analysis of Testees

**FIGURE 9:**
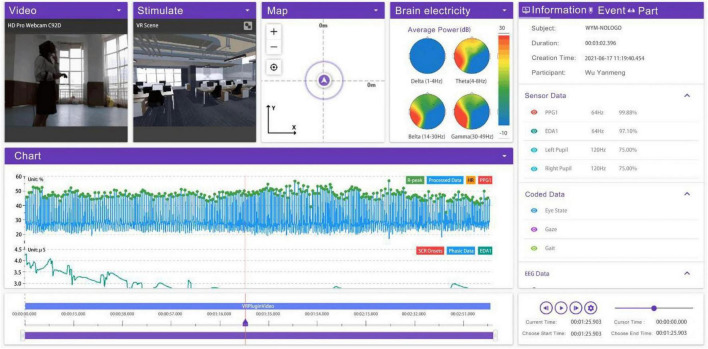
Experimental records of individual samples in ErgoLab (Search and take screenshots from ErgoLab by our research team).

During the experiment, we used the ErgoLab platform to analyze the eye movement data of the testees, mainly focusing on the number of blinks, the first fixation time (TFF) and the total fixation time (TFD) for comprehensive analysis. The figure (Figure 9) shows the eye movement data of the testee in the VR scene. The figure shows the conditions of the VR screens that the testee was concerned about at different time points of the test. The figure also shows the testee’s brainwave status, skin electrical status, and heart rate status in a real-time comprehensive display. We can judge whether the content of the testee concerned at that moment caused a comprehensive change in these data. In this way, we can judge which eye tracking data is true and reliable.

**FIGURE 10:**
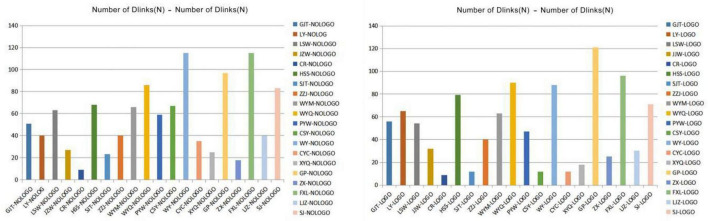
Statistics of the number of times the testees blink in scenes A and B (Search and take screenshots from ErgoLAB by our research team).

Number of blinks: By analyzing the number of blinks of the testees (Figure 10), we found that the overall average number of blinks of the 20 testees in scene A was 56 times. In scene B, the overall average number of blinks of the 20 testees was 51 times, indicating that the testees were more focused when observing scene B.

Time to first fixation (TFF): According to the derived data, the mission is the highest average among the five interest areas (community, belonging, mission, pride, and work fun) divided by 20 testees in the B scene Sensitive area, its TFF is 65S. The overall average time to fixation (TFF) is only 32.34S. Therefore, the first gaze time of the testee in the sense of mission is selected for research. The standard deviation of the TFF of the sense of mission is 60, which means that the individual differences are large.

Total fixation time (TFD): According to the derived data, among the five interest areas divided by the 20 testees in the B scene, the area with the highest total fixation time is also the sense of mission area, with a TFD of 6.19S. The overall total fixation time (TFD) average is only 2.01S. Therefore, by comparing with the first fixation time, we can find that the longer the testee’s first fixation time for the mission area in the corporate culture, the longer the overall fixation time.

### Interview Analysis of Testees

After the experience experiment of the virtual scene was completed, the research team conducted an interview with each testee. During the interview, 13 testees directly participated in the design business in this organization, and 7 testees participated in administrative or logistical duties related to the design business in this organization. All test testees are an important part of the staff in this organization. When asked what aspects of the company’s corporate culture the testee thinks include, some respondents said: “spatial layout,” “organizational slogan,” “flexible management,” “spatial theme,” “humanistic care,” “Open,” “tolerant,” “overtime,” “free,” “relaxed atmosphere,” and “artistic atmosphere.” When asked whether the testees think that a workplace full of corporate culture is more conducive to improving their work enthusiasm, more than half of the testees held a positive attitude. Some testees said that “may be beneficial to self-relaxation,” some said that this is related to whether the corporate culture fits their personal identity, and some said that “the atmosphere is more relaxed and work efficiency will increase.” When asked whether the testees prefer to work in space A (office space without corporate culture implantation) or space B (office space with corporate culture implantation) during the experiment, more than half of the testees I prefer space B. Most of the testees believe that allowing employees to see the corporate culture can increase enthusiasm and reduce stress. Seeing corporate culture in the space can make people impressed, happy, and help employees understand corporate culture. There are also testees who are more inclined to space A, the main reason is that “personally does not like the kind of corporate culture content posted on the wall.” One of the testees said that he didn’t like the two spaces, because the corporate culture content in the B space is too much, and there is no interesting and rich display form. He hopes to integrate into the corporate culture through simple space design techniques. He thinks that there is no display of corporate culture content in space A, and he doesn’t like it either. Through the interview, we can find three pieces of information. The first message is that most of the testees do not fully understand the culture of the organization, and the degree of understanding varies greatly. Therefore, there is a need to further promote the corporate culture within the organization. The second is that the information is that most of the testees have a positive attitude toward the view that a workplace full of corporate culture can enhance employees’ enthusiasm for work, so the corporate culture can be implanted in the office space to have a positive impact on employees. The third message is that office space with corporate culture implanted is easier for employees to accept, but the presentation form and method of corporate culture need to be considered reasonably.

### Questionnaire Analysis of Test Subjects

After the experiment, we also conducted a questionnaire survey on the testees. There are a total of 14 questions in the questionnaire, of which the first 10 questions are directly related to this research, and the last 4 questions are the personal information of the testees Appendix. In the survey that asked the testees to know the company’s corporate culture very well, 45% of the testees expressed “agree,” 50% of the testees expressed they were “unsure,” and 5% of the testees expressed “disagree.” This data shows that the participants’ awareness of the organization’s corporate culture has room for further improvement, and it is necessary to explore effective ways to improve employees’ understanding of corporate culture. In the survey that asked about the testees’ desire to incorporate relevant visual signs belonging to their company in the office space, 90% of the testees expressed “agree,” 5% of the testees expressed “disagree,” and 5% of the testees expressed “unsure.” This data shows that the testees have a higher acceptance of the integration of corporate visual identity into the office space. The presentation of corporate visual identity can become one of the important ways for corporate culture to be integrated into office space. In the survey that asked the testees that a high-quality office environment with a sense of design is conducive to stimulating their creativity, 95% of the testees expressed “agree,” and 5% of the testees expressed “unsure,” no testee expressed “disagree.” The data shows that most of the testees generally accept a high-quality office environment with a sense of design. They believe that the improvement of the environment can stimulate the creativity of employees. In the survey that asked the testees to think that an office space full of corporate culture is more conducive to improving their enthusiasm for work, 60% of the testees expressed “agree,” and 35% of the testees expressed “unsure,” 5% of the testees expressed “disagree.” This data shows that an office space full of corporate culture can have a positive impact on the motivation of most employees. In the survey that asked the testees that the office space full of corporate culture gave the testees a sense of belonging to the company, 75% of the testees expressed “agree,” and 20% of the testees expressed “unsure.” A 5% of the testees expressed “disagree.” This data shows that incorporating corporate culture into the office space can have a positive impact on the testee’s sense of belonging. In the survey that asked the testees that an office space full of corporate culture would enhance team cohesion, 80% of the testees expressed “agree,” 15% of the testees expressed “unsure,” and 5% of the testees expressed “disagree.” The data shows that incorporating corporate culture in the office space can have a positive impact on the cohesion of the testees’s team. In the survey that asked respondents to think that office spaces full of corporate culture should incorporate interesting spatial experiences, 95% of the testees expressed “agree,” and 5% of the testees expressed “unsure.” No testee expressed “disagree.” This data shows that interesting spatial experience is accepted by most people in the process of integrating corporate culture into office space. In the survey that asked the testees that an office space full of corporate culture should display the honors that the company has won, 60% of the testees expressed “agree,” 20% of the testees expressed “unsure,” and 20% of the testees expressed “disagree.” This data shows that it is accepted by most people to properly display the honors obtained by the company in the office space to convey the corporate culture. In the survey that asked the testees that an office space full of corporate culture is conducive to enhancing their sense of responsibility, 65% of the testees expressed “agree,” and 30% of the testees expressed “unsure.” A 5% of the testees expressed “disagree.” The data shows that integrating corporate culture into office space to enhance the sense of responsibility is acceptable to most of the testees. In the survey that asked the testees that it is necessary to present the development history of the company in the office space, 70% of the testees expressed “agree,” 20% of the testees expressed “unsure,” and 10% of the testees expressed “disagree.” This data shows that it is considered necessary for most of the testees to present the development memorabilia of the company in an office space full of corporate culture. Among those testees, the proportion of men was 65% and the proportion of women was 35%. The testees are all ordinary employees of the design, innovation and entrepreneurship organization, and all the testees have received a college degree or above. Through the analysis of the questionnaire, we found that the visual or spatial elements incorporated in the space B experimental scene are recognized by most of the testees.

### Discussion on the Data Set Used in the Research

The data set of this research is mainly composed of four parts of data. The data of these four parts are generated after analyzing the data collected by HME ErgoLAB Synchronization Cloud Platform. The data of these four parts are the physiological baseline data of all testees, the physiological data of the testees in two virtual scenes, the eye movement data of the testees in the experiment, the questionnaires and interview data of the testees. The physiological baseline data of the testees mainly includes the EDA data, EEG data and HRV data of the testees before entering the virtual scene experiment. These data are used to reflect the basic physiological data of all testees. The physiological data of the testees in the two virtual scenes mainly includes EDA data, EEG data and HRV data of all testees in the A scene and B scene experiments. These data are mainly used to reflect the changes in the physiological data of the testees during the experiment. The eye movement data of the testees in the experiment mainly includes the testees’ visual attention points, the number of blinks, the number of eye saccades, and the changes in pupil diameter during the experiment. These data are mainly used to reflect the visual attention changes of all testees in the virtual scene. The questionnaire and interview data of the testees mainly include the basic personal information data statistics of all the testees, the data statistics of the questionnaire fill-in results, and the relevant result statistics during the interview process. These data are mainly used to reflect the actual thoughts of the testees. These data can help illustrate the credibility of physiological data and eye movement data.

## Discussion of Related Research

The method of multi-channel physiological signal acquisition for analysis has been successfully applied in related research fields. Some researchers proposed in the paper to use psychophysiological methods to identify personal music emotional experience. In the experiment, researchers collected diverse information of EDA, PPG, SKT, RSP, and PD to explore personal music emotional experience. This research provides people with an effective way to identify personal music emotional experience (Zhang et al., 2019). And they also used the ErgoLAB platform in the experiment for synchronous comparative analysis of data. This experiment has shown that it is possible to distinguish individual psychological and emotional changes through diverse physiological data. Our research is also based on multi-channel physiological data collection. Not only that, we also collected EEG, HRV, and eye movement data, and the content of the data is more comprehensive. EEG and HRV can reflect people’s psychological changes. Some researchers collected brain activity, heart rate changes, and skin conduction data of participants in experiments to explore the psychological changes of people watching flowering plants in an office-like environment (Elsadek and Liu, 2020). This has important reference value for the establishment of our research database. In the research findings, they found that the blue and purple flowering plants in the office can give people a feeling of “comfort,” “relaxation,” and “cheerfulness.” This shows that in the office space, visual content can affect personal emotional changes. The visual presentation of corporate culture in the office space can also have a psychological impact on people. Using VR virtual scenes in the experiment can avoid the interference caused by other factors in the experiment. Some researchers use virtual reality technology to develop an immersive virtual museum in the research of indoor wayfinding to test the physiological and emotional responses of participants (Lin et al., 2019). However, they combined the VR virtual scene experiment with the participants’ EDA data and analyzed them. Based on this, our research has more diverse data analysis content. Some researchers use a variety of physiological data analysis combined with virtual scene experiments and eye movement analysis to evaluate architectural design goals in advance (Pei et al., 2021a). The difference between our experiments is that, based on the use of comprehensive data analysis, we incorporate the discussion of spatial narrative issues. Provide experimental basis for others to successfully carry out spatial narrative design behavior in office space.

## Conclusion

The spatial narrative of designing innovation and entrepreneurial organization culture is to integrate entrepreneurship and corporate culture symbols into the space through visual presentation. Disseminate unique corporate culture information through specific spatial visual logic. So as to achieve the use of space to “tell” entrepreneurial stories, cultivate entrepreneurial spirit, stimulate creativity, and generate positive organizational psychology. Through literature search, it is found that the research on the spatial field of design entrepreneurship is relatively in sufficient. Through the use of CiteSpace to draw a visual map, it is found that the relevant researchers have a low degree of closeness and less research frequency, and the research in this field is not sufficient. The cooperation status of related research institutions is scattered, and the research in this field is relatively weak. Around the core keywords of “entrepreneurship,” co-occurring keywords such as “Innovation,” “Organization,” “Technology,” and “Case study” appear, but they are insufficiently involved in psychology and there is a need for further research.

Whether the study of spatial narrative is successful needs to be judged by whether the change of people’s psychological cognition achieves the expected effect. The traditional qualitative research methods such as interviews and questionnaires cannot fully and accurately present the psychological cognitive mechanism of design innovation and entrepreneurial organization members. It is necessary to explore the subtle changes between the spatial narrative of design innovation and entrepreneurship organizational culture and the psychological cognition of innovation and entrepreneurship through scientific quantitative experiments. Through multi-dimensional objective and quantitative experimental data analysis, combined with the process of qualitative research, we make the research method more scientific and comprehensive. Our exploration in research methods makes up for the lack of precise quantitative research in this field. We use ErgoLAB HME Synchronization Technology to collect the physiological data, EEG data and eye movement data of the testees to realize the real-time synchronous collection and analysis of multi-dimensional data. The hypothesis proposed in this research is that the implantation of innovative and entrepreneurial organizational culture has a positive impact on people’s psychological cognition in the process of spatial narration. Through this research, we have revealed the subtle changes between the spatial narrative and individual psychological cognition about the design and innovation of entrepreneurial organizational culture. We have confirmed through experiments that the implantation of entrepreneurial organizational culture has a positive impact on people’s psychological cognition in the process of spatial narration. An office space that incorporates design innovation and entrepreneurial organizational culture is conducive to inspiring employees’ sense of belonging, collective sense, pride, mission, and work fun. Through the use of virtual reality imaging technology combined with electrophysiological technology to carry out quantitative experiments on the psychological cognition of participants in the spatial narrative of design innovation and entrepreneurial organizational culture. Physiological indicators, EEG indicators and eye movement indicators are important quantitative indicators in this study. According to the data obtained in this experiment, we analyzed from the eye tracking data and found that in a scene with elements related to corporate organizational culture of design innovation and entrepreneurial organizations, the first fixation time and total fixation time of the relevant cultural elements in the area are correlated. The testees can pay attention to the design of the interest partition in the scene. From the analysis of physiological and EEG data, it is found that the mentality of the testees when watching the two scenes is relatively stable, while in scene B, the average heart rate and LF/HF values of the subjects have increased. The testees are more autonomous when observing scenes with corporate culture space. Combining the testee’s brainwave alpha wave data and beta wave data, it is found that the comfort and excitement of the testee in scene B are better. Combined with the interviews, we found that most of the testees did not fully understand the organization’s culture, and the degree of understanding differed greatly. There is a need to further promote the corporate culture. Most of the testees hold a positive attitude toward the view that a workplace full of corporate culture can enhance employees’ enthusiasm for work, so the corporate culture can be implanted in the office space to have a positive impact on employees. Office space with corporate culture implanted is easier for employees to accept, but the presentation form and method of corporate culture need to be considered reasonably. Through questionnaire analysis, we found that the visual or spatial elements incorporated in experimental scene B are recognized by most of the subjects. The results of quantitative analysis, interview analysis, and questionnaire survey all show that the evaluation of scenario B is better, indicating that the results of the quantitative and quantitative analysis of this study are consistent. Most of the testees generally accept a high-quality office environment with a sense of design, and believe that the improvement of the environment can stimulate the creativity of employees. An office space full of corporate culture can have a positive impact on the work enthusiasm, sense of belonging and team cohesion of most employees. The results of this experiment provide a data reference for stimulating the innovation ability of members in innovative and entrepreneurial organizations. People who design innovative and entrepreneurial organizations can use these dimensions to carry out spatial narration in the workspace, and to achieve the harmony and unity of people, culture, and environment. In the future, we will further discuss the quantitative impact of different design innovations and entrepreneurial organizational cultures and more spatial narrative forms on psychological cognition, and continue to improve data quantitative indicators and establish a quantitative evaluation mechanism.

## Data Availability Statement

The raw data supporting the conclusions of this article will be made available by the authors, without undue reservation.

## Ethics Statement

Ethical review and approval were not required for the study on human participants in accordance with the local legislation and institutional requirements. Written informed consent was obtained from the individual(s) for the publication of any potentially identifiable images or data included in this article.

## Author Contributions

JH was mainly responsible for experiment organization, execution, and result analysis. XZ was mainly responsible for the communication, experimental design, and data search for this study. Both authors contributed to the article and approved the submitted version.

## Conflict of Interest

The authors declare that this research is supported by Shanghai Fengyuzhu Culture Technology Co., Ltd. The funder was not involved in the study design, collection, analysis, interpretation of data, the writing of this article or the decision to submit it for publication.

## Publisher’s Note

All claims expressed in this article are solely those of the authors and do not necessarily represent those of their affiliated organizations, or those of the publisher, the editors and the reviewers. Any product that may be evaluated in this article, or claim that may be made by its manufacturer, is not guaranteed or endorsed by the publisher.

## Footnotes

1 The platform has synchronized heart rate variability data analysis (HRV), electroencephalogram data analysis (EEG), skin conductance data analysis (EDA), electromyography data analysis (EMG), behavior coding, motion capture, eye-tracking, spatial-temporal trajectory And functional modules such as interaction analysis and sequential statistical analysis.

## Appendix

### Attachment to the paper

Psychological research on the integration of the companyz’s organizational culture into the office space

Dear friends:

Thank you very much for participating in this questionnaire in your busy schedule! The purpose of this survey is to understand whether the integration of the design innovation and entrepreneurial organizational culture in the work space has a positive impact on the psychology of the team’s employees. We assure you that the information obtained in this questionnaire is limited to academic research, please follow your real ideas Fill in, thank you very much for your support and cooperation!

1. Do you have a good understanding of the organizational culture of this company?
    **A.** Agree **B.** Is not sure **C.** Disagree
2. Do you want to incorporate relevant visual signs belonging to your company in your office space?
    **A.** Agree **B.** Is not sure **C.** Disagree
3. Do you think that a high-quality office environment with a sense of design is conducive to stimulating your creativity?
    **A.** Agree **B.** Is not sure **C.** Disagree
4. Do you think that an office space full of organizational culture is more conducive to enhancing your enthusiasm for work?
    **A.** Agree **B.** Is not sure **C.** Disagree
5. Do you think that office space full of organizational culture gives you a sense of corporate belonging?
    **A.** Agree **B.** Is not sure **C.** Disagree
6. Do you think an office space full of organizational culture will enhance team cohesion?
    **A.** Agree **B.** Is not sure **C.** Disagree
7. Do you think that an office space full of organizational culture should incorporate interesting spatial experience?
    **A.** Agree **B.** Is not sure **C.** Disagree
8. Do you think an office space full of organizational culture should show the honors the company has won?
    **A.** Agree **B.** Is not sure **C.** Disagree
9. Do you think that an office space full of organizational culture is conducive to enhancing your sense of responsibility?
    **A.** Agree **B.** Is not sure **C.** Disagree
10. Do you think it is necessary to present the development memorabilia of the company in the office space?
    **A.** Agree **B.** Is not sure **C.** Disagree
11. Your gender:  □ Female □ Male
12. Your job title: □ Management (director, project manager, mid-level company, senior company executives, etc.)
           □ General staff
13. Your age range:
    □ Born in 2000–2010
    □ Born in 1990–1999
    □ Born in 1980–1989
    □ Born in 1970–1979
    □ Born in 1969 or before
14. Your education:
    □ Three-year university diploma
    □ Undergraduate Diploma
    □ Master’s Degree
    □ Doctor’s degree
